# Genetic Factors of Teeth Impaction: Polymorphic and Haplotype Variants of *PAX9*, *MSX1*, *AXIN2*, and *IRF6* Genes

**DOI:** 10.3390/ijms241813889

**Published:** 2023-09-09

**Authors:** Grzegorz Trybek, Aleksandra Jaroń, Ewa Gabrysz-Trybek, Monika Rutkowska, Aleksandra Markowska, Krzysztof Chmielowiec, Jolanta Chmielowiec, Anna Grzywacz

**Affiliations:** 1 Department of Oral Surgery, Pomeranian Medical University in Szczecin, Powstańców Wielkopolskich 72/18, 70-111 Szczecin, Poland; 2 4th Military Clinical Hospital in Wroclaw, ul. Rudolfa Weigla 5, 50-981 Wroclaw, Poland; jaronola@gmail.com (A.J.); olka.markowska@gmail.com (A.M.); 3 Individual Specialist Medical Practice Ewa Gabrysz-Trybek, 70-111 Szczecin, Poland; ewa_gabrysz@wp.pl; 4 Department of Hygiene and Epidemiology, Collegium Medicum, University of Zielona Góra, 28 Zyty St., 65-046 Zielona Góra, Poland; chmiele@vp.pl (K.C.); chmiele1@o2.pl (J.C.); 5 Independent Laboratory of Health Promotion, Pomeranian Medical University in Szczecin, Powstańców Wielkopolskich 72 St., 70-111 Szczecin, Poland; grzywacz.anna.m@gmail.com

**Keywords:** *MSX1*, *PAX9*, *AXIN2*, *IRF6*, genetic phenomenon, genetic association studies, tooth, impacted, odontogenesis, polymerase chain reaction, nucleotides

## Abstract

In recent research, there has been a growing awareness of the role of genetic factors in the positioning and eruption of teeth in the maxilla and mandible. This study aimed to evaluate the potential of specific polymorphic markers of single nucleotide polymorphisms (SNPs) located within the *PAX9*, *MSX1*, *AXIN2*, and *IRF6* genes to determine the predisposition to tooth impaction. The study participants were divided into two groups: the first group consisted of individuals with at least one impacted secondary tooth. In contrast, the second group (control group) had no impacted teeth in their jaws. To analyze the genes, real-time PCR (polymerase chain reaction) and TaqMan probes were utilized to detect the selected polymorphisms. The findings suggest that disruptions in the structure and function of the mentioned genetic factors such as polymorphic and haplotype variants of *PAX9*, *MSX1*, *AXIN2*, and *IRF6* genes, which play a direct role in tooth and periodontal tissue development, might be significant factors in tooth impaction in individuals with genetic variations. Therefore, it is reasonable to hypothesize that tooth impaction may be influenced, at least in part, by the presence of specific genetic markers, including different allelic variants of the *PAX9*, *AXIN2*, and *IRF6* genes, and especially *MSX1*.

## 1. Introduction

Extensive research has revealed that tooth development in humans and other mammals is influenced by both genetic and environmental factors [1,2,3]. Interactions between hundreds of genes control the number, shape, and location of teeth during embryogenesis [1,2,3]. The genetic basis of tooth developmental disorders, including agenesis, extra teeth, and structural anomalies, has been extensively documented [4,5,6,7]. Specific genetic factors have also been associated with abnormalities in tooth location and eruption [7]. Additionally, the impact of genetical, pre-, peri-, and postnatal risk factors on enamel development disorders should be considered. These risk factors include severe illness or complications during pregnancy, low birth weight or premature birth, breastfeeding issues or illness, and early childhood infections [8].

Studies in animals have shown that dental defects, including impacted teeth, are often influenced by multiple genes [9,10]. Currently, an impacted tooth is defined as a fully developed tooth embedded in the jawbone or mandible, with closed and mineralized root apices. It can be categorized as complete if fully covered by bone, or partial if the crown has perforated the bone plate and remains adjacent to the soft tissues. Impacted teeth no longer exhibit growth tendencies [11,12].

Researchers are continuously delving into the intricate relationship between genetic and environmental factors in tooth development, advancing our understanding of dental defects and their underlying mechanisms. Impacted teeth are commonly found in the maxilla, specifically the canines and incisors, as well as in the mandible, including the third molars and second premolars. The prevalence of impacted teeth in the anterior region ranges from 0.9% to 4.3% of the population, with canines accounting for 70% and incisors for 11.5% of cases [13,14]. The diagnosis of lower third molar impaction varies across populations and time periods. In the 1960s, the prevalence ranged from 17% to over 70% among young Europeans, while more recent studies in the early 2000s have reported varying figures [15].

Extensive research on the genetic basis of dental disorders has identified several candidate genes, such as *MSX1*, *PAX9*, *AXIN2*, WNT10A, EDA, EDAR, BMP4, FGF8, *IRF6*, and PITX2 [16,17,18,19,20,21,22,23,24,25,26]. These genes encode proteins with diverse functions, including signaling molecules, receptors, mediators, and gene expression regulators. They participate in various signaling pathways, including WNT/β-catenin, transforming growth factor-beta (TGFβ), and BMP pathways [16]. Mutations in the *MSX1* and *PAX9* genes are frequently associated with tooth developmental disorders. These genes encode transcriptional regulators that play a crucial role in tooth formation and the development of other structures [1,2]. The *PAX9* gene has been extensively studied in terms of its molecular structure, expression, and potential effects on tooth development processes [27,28,29,30].

The *PAX9* gene, located on chromosome 14 in humans, is linked to various dental abnormalities. It consists of four exons, with the first exon acting as a start codon and exon 2 encoding a highly conserved region of the pairing domain [31]. Most mutations in the *PAX9* gene have been identified in exon 2, or less frequently, in exon 4. These mutations are associated with different forms of agenesis, non-syndromic hypodontia, and oligodontia [29,32,33]. Specific alleles at polymorphic sites in exon 2 or 4, as well as the deletion of one copy of the *PAX9* gene, can lead to selective tooth agenesis, particularly in molars. The observed phenotype is often seen in heterozygotes, indicating that haploinsufficiency of the *PAX9* gene contributes to tooth agenesis [30,34].

The *MSX1* gene, located on chromosome 4 in humans, consists of two exons, 2332 and 1229 bp in length, separated by a 2332 bp intron. It produces two distinct transcripts, resulting in two polypeptides of different lengths of 303 and 297 amino acids [35,36]. The transcription factor encoded by the *MSX1* gene regulates the expression of multiple target genes and interacts with proteins involved in odontogenesis, such as *PAX9*, Dlx family proteins, and Tbp [37]. Genes like *MSX1*, which code for proteins containing a homeodomain, play a crucial role in odontogenesis, controlling various stages of tooth development and influencing the final dentition pattern [38]. The *AXIN2* gene is responsible for encoding a factor in the WNT signaling pathway, which regulates the stability of beta-catenin. Disruptions in this pathway can contribute to the development of certain neoplasms [39,40,41]. The WNT pathway controls the levels and localization of beta-catenins. Activation of the WNT-dependent pathway stabilizes beta-catenin, allowing it to bind to transcription factors from the TCF family and regulate target gene expression. In the absence of WNT signals, beta-catenin undergoes phosphorylation and degradation through a multiprotein complex involving the APC protein, *AXIN1*, and *AXIN2* proteins [42,43,44,45]. AXIN proteins, including *AXIN2*, facilitate the formation of beta-catenin-degrading complexes [46]. *AXIN2* gene expression is induced by the WNT signaling pathway, indicating that *AXIN2* acts as a negative regulator in a feedback loop [47,48]. The SHH and WNT pathways, along with proteins like *MSX1* and *PAX9*, play crucial roles in normal tooth morphogenesis and contribute to the complex regulation of tooth development [49,50]. The *IRF6* gene is associated with van der Woude syndrome (VWS) and Bartsocas–Papas congenital anomaly syndrome. VWS is characterized by cleft lip and palate, mucous cysts of the lower lip, and tooth agenesis [51]. Certain SNP mutations in the *IRF6* gene have been significantly correlated with the presence of cleft lip with or without cleft palate (CL/P) in various populations [52,53,54,55,56,57]. The *IRF6* gene may also contribute to tooth developmental disorders, especially when other craniofacial abnormalities are present. The development of teeth, palate, and lips is interconnected, and the severity of cleft symptoms is often associated with the number of affected teeth [58,59,60,61,62,63,64,65,66,67]. Therefore, the *IRF6* gene is considered important in the study of dental development disorders due to its confirmed role in the etiology of cleft lip and palate.

This research’s main objective was to assess the usefulness of selected polymorphic markers of single nucleotide polymorphism (SNP) characters located within the *PAX9*, *MSX1*, *AXIN2*, and *IRF6* genes in the context of their applicability to determine the predisposition to the occurrence of the tooth impaction phenomenon. The protein products of the *PAX9*, *MSX1*, *AXIN2*, and *IRF6* genes directly participate in the processes of tooth formation. Therefore, it has been hypothesized that the polymorphism of these genes may contribute to the phenomenon of impacted teeth in humans.

## 2. Results

Table 1, Table 2, Table 3 and Table 4 describe the frequency of impaction of different groups of teeth in the maxilla and mandible.

Differences in the frequency of the *MSX1 rs8670* gene polymorphism were found in respondents with impacted teeth between maxilla and mandible(^max&man^) together, and maxilla(^max^) and mandible(^man^) separately (C/C^max&man^ 0.47 vs. C/C^max^ 0.71 vs. C/C^man^ 0.34; T/T^max&man^ 0.05 vs. T/T^max^ 0.05 vs. T/T^man^ 0.14; C/T^max&man^ 0.48 vs. C/T^max^ 0.24 vs. C/T^man^ 0.24; χ^2^ = 15.876; *p* = 0.0031, Table 4).

Differences in the frequency of the *MSX1 rs12532* gene polymorphism were found in respondents with impacted teeth between maxilla and mandible (^max&man^) together, and maxilla(^max^) and mandible(^man^) separately (A/A^max&man^ 0.61 vs. A/A^max^ 0.29 vs. A/A^man^ 0.58; G/G^max&man^ 0.06 vs. G/G^max^ 0.21 vs. G/G^man^ 0.08; A/G^max&man^ 0.33 vs. A/G^max^ 0.50 vs. A/G^man^ 0.34; χ^2^ = 15.1036; *p* = 0.0044) and alleles (A^max&man^ 0.78 vs. A^max^ 0.54 vs. A^man^ 0.75; G^max&man^ 0.22 vs. G^max^ 0.46 vs. G^man^ 0.25; χ^2^ = 16.3635; *p* = 0.0003) (Table 4).

Differences in the frequency of the *AXIN2 rs2240308* gene polymorphism were found in respondents with impacted teeth between maxilla and mandible (^max&man^) together, and maxilla (^max^) and mandible(^man^) separately (A/A^max&man^ 0.30 vs. A/A^max^ 0.13 vs. A/A^man^ 0.40; G/G^max&man^ 0.21 vs. G/G^max^ 0.39 vs. G/G^man^ 0.17; A/G^max&man^ 0.49 vs. A/G^max^ 0.47 vs. A/G^man^ 0.43; χ^2^ = 11.2104; *p* = 0.0243) and alleles (A^max&man^ 0.55 vs. A^max^ 0.37 vs. A^man^ 0.61; G^max&man^ 0.45 vs. G^max^ 0.63 vs. G^man^ 0.39; χ^2^ = 11.3513; *p* = 0.00343) (Table 4).

The *PAX9 rs4904210*, *AXIN2 rs7591*, *AXIN2 rs4904210*, *IRF6 rs642961*, *IRF6 rs861019*, *IRF6 rs4904210*, and *IRF6 rs658860* genotype and allele frequencies in the studied sample did not differ in respondents with impacted teeth between maxilla and mandible together, and maxilla and mandible separately (Table 4).

For genotypes and alleles for which statistical significances were demonstrated, a comparison of allele and genotype frequencies between the study and control groups is also presented in Table 5.

## 3. Discussion

When examining the phenotypic impact of mutations, certain circumstances can associate an allele reducing functional protein levels with favorable traits. For instance, the reduction in C-allele teeth at the rs4904210 locus in the *PAX9* gene, primarily affecting the development of the third molars, has been observed in studies and considered not only neutral but even beneficial [68].

The shift in modern lifestyles, with a preference for highly processed and cooked foods over the raw and hard diet of our ancestors, has rendered third molars unnecessary remnants of our species’ evolutionary history [69]. In cases of impacted or abnormally erupted molars, their presence can pose health hazards, serving as sites for inflammation and bacterial impaction, leading to cavities in neighboring teeth and infections in the bone tissue, potentially weakening or even destroying them. Additionally, bacteria multiplying in these areas can spread systemically, attacking other organs and posing health risks [70].

In this context, the Ala240Pro mutation in the *PAX9* gene is described by some authors as a beneficial change aimed at eliminating unnecessary third molars from the modern human population [68].

Functional analysis of the Arg196Pro polymorphism in the *MSX1* gene, involved in hypodontia, showed that the structurally defective protein with reduced thermal stability resulted in impaired DNA binding and protein interactions. This mutation was associated with a higher frequency of missing second premolars and third molars, and in some cases, upper premolars and lower first molars [71].

In a study of the Chinese population, a new C662A mutation in the *MSX1* gene’s homeodomain region was identified, leading to an Ala221Glu substitution. This mutation disrupts the binding to DNA and interactions with other proteins, such as those from the DLX family, hindering the initiation of epithelial–mesenchymal signal cascades during tooth development [72]. Carriers of the Ala221Glu mutation exhibited selective tooth loss, including premolars, third molars, lateral incisors, and canines [73].

Another mutation in the *MSX1* gene’s homeodomain region is the T671C substitution, resulting in a Leu224Pro substitution. Predictive analysis suggests that this mutation may completely abolish the activity of the *MSX1* protein, leading to the congenital absence of second premolars and third molars in carriers [74].

Bearing in mind the information presented above on the influence of various mutations in the coding area of the *MSX1* gene homeodomain, it is worth emphasizing that the functional analysis of the remaining five missense mutations in the same region of the *MSX1* gene, correlated with hypodontia (Met61Lys, Ala194Val, Arg196Pro, Ala219Thr, and Ala221Glu), revealed none of the previously known molecular mechanisms (such as a disturbance of the interaction of *MSX1* with *PAX9* or other regulatory proteins). Considering the influence of various mutations in the coding region of the *MSX1* gene homeodomain, it is important to note that functional analysis of five missense mutations (Met61Lys, Ala194Val, Arg196Pro, Ala219Thr, and Ala221Glu) associated with hypodontia revealed that previously known molecular mechanisms could not fully explain the significance of these amino acid substitutions in tooth agenesis [22].

Furthermore, it was demonstrated that most of these missense mutations did not impair the crucial interaction between *MSX1* and *PAX9*, which activates the expression of the BMP4 gene, suggesting that the role of *MSX1* in tooth development is not directly linked to the interaction with *PAX9* [22]. This implies that potential molecular targets responsible for dental abnormalities may lie in other regions of the *MSX1* gene beyond the homeodomain.

Promising variants of the *MSX1* gene, relevant to various dental development disorders, include polymorphic sites rs8670 and rs12532, located in the non-coding UTR region of exon 2 [75]. These variants were found in the Polish population and primarily associated with non-syndromic sporadic oligodontics in humans [76].

The non-coding regions of the *MSX1* gene may also contribute to the development of dental disorders, independent of the homeodomain’s direct functioning. The rs8670 variant in the *MSX1* gene was found to potentially correlate with non-syndromic hypodontia in the upper lateral incisors, suggesting an impact on mRNA stability, antisense RNA-mediated interactions, or translation processes [77]. However, larger studies in Brazilian patients did not show significant differences in the distribution of genotypes and alleles in the rs8670 and rs12532 sites between the agenesis group and the control group [78].

In patients with severe familial hypodontia, direct sequencing of the *AXIN2* gene revealed a nonsense mutation (Arg656Stop) in exon 7, which led to premature translation termination [79]. This mutation may result in mRNA degradation through the nonsense-mediated decay mechanism, as the mRNA transcript with a premature stop codon fulfills the conditions for degradation [80,81]. The protein produced in individuals with the C1966T mutation lacks the C-terminal domain responsible for oligomerization and interaction with other proteins [42,82,83]. Consequently, individuals with this mutation experience a decrease in the function of the *AXIN2* protein, leading to the accumulation of beta-catenin [84]. Sequence analysis of the *AXIN2* gene in Polish patients with selective tooth agenesis identified several polymorphic sites, including two new variants in the intron region and one variant in exon 7 [85]. Statistical analysis revealed that carriers of the c.956 + 16G and c.2062T alleles had an increased risk of selective tooth agenesis, indicating a potential association [85]. The synonymous change Leu688Leu may disrupt the exonic splicing enhancer sequence, affecting the recognition of cleavage sites in splicing processes [81]. The c.956 + 16A>G mutation could also impact splicing processes, creating an additional donor site within exon 2, potentially interfering with odontogenesis [85].

In studies involving Brazilian and Turkish patients with selective tooth agenesis, three intragenic polymorphic sites (rs7591, rs11867417, and rs2240308) in the *AXIN2* gene were analyzed [86]. The presence of the rs2240308 variant, either in isolation or as part of a haplotype, correlated with the absence of at least one incisor in the studied populations [86]. Although in silico analysis did not confirm this variant as a direct etiological factor, it is suggested that it may interact with another variant to contribute to the development of tooth developmental disorders [86].

Associations between selected markers (rs7802, rs861019, and rs2235371) within the *IRF6* gene and preferential agenesis of premolars were confirmed in the Brazilian population [87]. The non-synonymous mutation rs2235371 (Val274Ile) was found to have an attributable fraction (AF) coefficient value of 16.4%, indicating its contribution to agenesis cases [87]. Haplotypes reconstructed for the analyzed polymorphic sites in the *IRF6* gene, containing the more common allele at rs2235371, were consistently found in agenesis patients, particularly in relation to premolar agenesis [87].

However, it was suggested that the rs2235371 variant may not be an etiological factor in itself, but rather interacts with other variants that directly impact the appearance of agenesis [88]. To further investigate, additional markers within the *IRF6* gene (rs4844880, rs2235371, rs2013162, rs861019, rs2073487, rs642961, and rs658860) were analyzed. The presence of rs658860 variants and the haplotype [A; T; A] reconstructed for sites [rs861019; rs2073487; rs642961] showed a correlation with abnormal development of at least one incisor [88].

In patients with impaired premolar development, the association was observed for the rs2013162 and rs642961 variants, as well as the haplotypes [G; T; A] in [rs861019; rs2073487; rs642961] and [T; AND; T] in [rs2073487; rs642961; rs658860] [88]. The presence of the rs642961 site in all statistically significant haplotype systems suggests its potential functional role. In silico analyses indicate that the rs642961 mutation may alter the AGAAT sequence near the *IRF6* gene to the AGGAT sequence, which is responsible for binding to the product of the heat shock transcription factor (HSF) gene, a transcription factor activated in response to temperature stress. Additionally, the rs861019 mutation was found to cause a change in another sequence (GTTCT → ATTCT) within the HSF binding domain [88]. However, the precise mechanism by which disruption of HSF binding affects normal tooth development remains unknown.

## 4. Materials and Methods

### 4.1. Subjects

Participation in the research project was offered to a consecutive group of 392 patients who visited the Department of Oral Surgery. The inclusion criteria for the study group required the presence of at least one fully impacted tooth in either the maxillary or mandibular bone. An impacted tooth was defined as a tooth surrounded by bone in X-ray images but absent in the dental arch during eruption. The study group consisted of 204 individuals, including 82 males and 122 females, who had one to seven impacted teeth. Patients under the age of sixteen and those who were unable to undergo a panoramic radiograph or had multiple missing teeth that prevented a diagnosis of hypodontia were not eligible for the project. Relatives of the patients were also excluded.

The study participants were divided into two groups based on the presence or absence of a diagnosis of tooth/teeth impaction provided by a medical specialist:

The study group included patients with at least one permanently impacted tooth in the maxillary or mandibular bone, as well as patients with supernumerary teeth.

The control group consisted of patients who had no impacted teeth in the maxillary bones and whose permanent teeth had properly emerged and aligned in the dental arches.

All patients were fully informed about the purpose, benefits, and research methods of the study. After providing written consent to participate, they were assigned individual identification numbers to ensure anonymity.

The study group included 204 patients (82 males and 122 females) in whom one to seven impacted teeth were identified (median 3, interquartile range 2–4). A histogram of patients in the study group with a specific number of impacted teeth is shown in Table 5. The most significant number of patients in the study group were observed to have four impacted teeth, while patients with two impacted teeth were the second most numerous subgroup.

It was assumed that the size of the Phi effect would be 0.25 with a group of 204 tested people, and the power of the statistical test for the polymorphism of the tested genes would be 0.83. However, for alleles (*n* = 408) the Phi effect size was assumed to be 0.16 and the power of the statistical test to be 0.83. The actual results obtained for the size of the Phi effect and the power of the statistical test are presented in Table 6.

### 4.2. Genotyping

Genomic DNA was extracted from venous blood using standard procedures. Genotyping was conducted with the real-time PCR method.

LightCycler^®^ 480 II System (Roche Diagnostic, Basel, Switzerland) was applied to perform the fluorescence resonance energy into the genotypic data. The data relating to the DRD2 gene polymorphism were obtained under the following conditions: PCR was performed with 50 ng DNA of each sample in a final volume of 20 µL containing 2 µL reaction mix, 0.5 mM of each primer, 0.2 mM of each hybridization probe, and 2 mM MgCl2 according to the manufacturer’s instructions with initial denaturation (95 °C for 10 min) and then 35 cycles of denaturation (95 °C for 10 s), annealing (60 °C for 10 s), and extension (72 °C for 15 s). After amplification, a melting curve was generated by holding the reaction at 40 °C for 20 s and heating slowly to a level of 95 °C. The fluorescence signal was plotted against temperature to provide melting curves for each sample.

### 4.3. Statistical Analysis

The frequencies of genotypes and alleles of the *PAX9 rs4904210*, *MSX1 rs8670*, *MSX1 rs12532*, *AXIN2 rs7591*, *AXIN2 rs4904210*, *AXIN2 rs2240308*, *IRF6 rs642961*, *IRF6 rs861019*, *IRF6 rs4904210*, and *IRF6 rs658860* polymorphisms in analyzed groups were compared with the chi-square test. All analyses were performed using STATISTICA 13 (Tibco Software Inc., Palo Alto, CA, USA) for Windows (Microsoft Corporation, Redmond, WA, USA).

## 5. Conclusions

Abnormalities in the structure and function of the previously described genetic factors directly involved in the development of the tooth, including its hard tissues and periodontal tissues, may therefore have a role in the appearance of retained teeth in individuals with such alterations in genetic material. On the basis of this study, it can be assumed that the impaction of teeth will also depend, at least in part, on the presence of certain genetic markers, particularly *AXIN2 rs2240308* and *MSX1 rs12532*.

## Figures and Tables

**Table 1:** Number of impacted molars in maxilla and mandible together, and in maxilla and mandible separately in respondents with impacted teeth.

Location of Impacted Teeth	Impacted Molars—0 *n* (%)	Impacted Molars—1 *n* (%)	Impacted Molars—2 *n* (%)	Impacted Molars—3 *n* (%)	Impacted Molars—4 *n* (%)	Impacted Molars—5 *n* (%)	Ʃ
Maxilla and mandible	1 (0.01)	1 (0.01)	15 (0.14)	24 (0.23)	64 (0.60)	1 (0.01)	106
Maxilla	10 (0.26)	13 (0.34)	5 (0.13)	2 (0.05)	8 (0.21)	0 (0.00)	38
Mandible	3 (0.05)	17 (0.28)	37 (0.62)	0 (0.00)	3 (0.05)	0 (0.00)	60
Ʃ	14	31	57	26	75	1	204

**Table 2:** Number of impacted premolars in maxilla and mandible together, and in maxilla and mandible separately in respondents with impacted teeth.

Location of Impacted Teeth	Impacted Premolars—0 *n* (%)	Impacted Premolars—1 *n* (%)	Impacted Premolars—2 *n* (%)	Impacted Premolars—5 *n* (%)	Ʃ
Maxilla and mandible	103 (0.97)	2 (0.02)	0 (0.00)	1 (0.01)	106
Maxilla	37 (0.97)	0 (0.00)	1 (0.03)	0 (0.00)	38
Mandible	57 (0.95)	3 (0.05)	0 (0.00)	0 (0.00)	60
Ʃ	197	5	1	1	204

**Table 3:** Number of impacted canines in maxilla and mandible together, and in maxilla and mandible separately in respondents with impacted teeth.

Location of Impacted Teeth	Impacted Canines—0 *n* (%)	Impacted Canines—1 *n* (%)	Impacted Canines—2 *n* (%)	Ʃ
Maxilla and mandible	103 (0.97)	3 (0.03)	0 (0.00)	106
Maxilla	26 (0.68)	11 (0.29)	1 (0.03)	38
Mandible	59 (0.98)	1 (0.02)	0 (0.00)	60
Ʃ	188	15	1	204

**Table 4:** Frequency of genotypes and alleles of the *PAX9 rs4904210*, *MSX1 rs8670*, *MSX1 rs12532*, *AXIN2 rs7591*, *AXIN2 rs4904210*, *AXIN2 rs2240308*, *IRF6 rs642961*, *IRF6 rs861019*, *IRF6 rs4904210*, and *IRF6 rs658860* polymorphisms in respondents with impacted teeth in maxilla and mandible together, and maxilla and mandible separately.

Location of Impacted Teeth	Genotypes			Alleles	
	*PAX9 rs4904210*				
	GG *n* (%)	CC *n* (%)	GC *n* (%)	G *n* (%)	C *n* (%)
Maxilla and mandible *n* = 106	42 (0.40)	15 (0.14)	49 (0.46)	133 (0.63)	79 (0.37)
Maxilla *n* = 38	19 (0.50)	7 (0.18)	12 (0.32)	50 (0.66)	26 (0.34)
Mandible *n* = 58	20 (0.34)	11 (0.19)	27 (0.47)	67 (0.58)	49 (0.42)
χ^2^ *p*-value φ Statistical test power	3.592 0.4640 0.13 0.28			1.394 0.4982 0.06 0.17	
	*MSX1 rs8670*				
	CC *n* (%)	TT *n* (%)	CT *n* (%)	C *n* (%)	T *n* (%)
Maxilla and mandible *n* = 106	50 (0.47)	5 (0.05)	51 (0.48)	151 (0.71)	61 (0.29)
Maxilla *n* = 38	27 (0.71)	2 (0.05)	9 (0.24)	63 (0.83)	13 (0.17)
Mandible *n* = 58	36 (0.62)	8 (0.14)	14 (0.24)	86 (0.74)	30 (0.26)
χ^2^ *p*-value φ Statistical test power	15.876 * 0.0031 0.28 0.91			3.986. 0.1363 0.10 0.42	
	*MSX1 rs12532*				
	AA *n* (%)	GG *n* (%)	AG *n* (%)	A *n* (%)	G *n* (%)
Maxilla and mandible *n* = 106	65 (0.61)	6 (0.06)	35 (0.33)	165 (0.78)	47 (0.22)
Maxilla *n* = 38	11 (0.29)	8 (0.21)	19 (0.50)	41 (0.54)	35 (0.46)
Mandible *n* = 59	34 (0.58)	5 (0.08)	20 (0.34)	88 (0.75)	30 (0.25)
χ^2^ *p*-value φ Statistical test power	15.104 * 0.0044 0.27 0.89			16.364 * 0.0003 0.20 0.96	
	*AXIN2 rs7591*				
	TT *n* (%)	AA *n* (%)	TA *n* (%)	T *n* (%)	A *n* (%)
Maxilla and mandible *n* = 106	44 (0.42)	19 (0.18)	43 (0.41)	131 (0.62)	81 (0.38)
Maxilla *n* = 38	7 (0.18)	9 (0.24)	22 (0.58)	36 (0.47)	40 (0.53)
Mandible *n* = 59	20 (0.33)	14 (0.23)	26 (0.43)	66 (0.56)	52 (0.44)
χ^2^ *p*-value φ Statistical test power	7.013 0.1352 0.185 0.54			4.904 0.0861 0.11 0.50	
	*AXIN2 rs4904210*				
	CC *n* (%)	TT *n* (%)	CT *n* (%)	C *n* (%)	T *n* (%)
Maxilla and mandible *n* = 106	55 (0.52)	11 (0.10)	40 (0.38)	150 (0.71)	62 (0.29)
Maxilla *n* = 38	13 (0.34)	8 (0.21)	17 (0.45)	43 (0.57)	33 (0.43)
Mandible *n* = 60	30 (0.50)	7 (0.12)	23 (0.38)	83 (0.69)	37 (0.31)
χ^2^ *p*-value φ Statistical test power	4.833 0.3050 0.15 0.36			5.316 0.0701 0.11 0.50	
	*AXIN2 rs2240308*				
	AA *n* (%)	GG *n* (%)	AG *n* (%)	A *n* (%)	G *n* (%)
Maxilla and mandible *n* = 106	32 (0.30)	22 (0.21)	52 (0.49)	116 (0.55)	96 (0.45)
Maxilla *n* = 38	5 (0.13)	15 (0.39)	18 (0.47)	28 (0.37)	48 (0.63)
Mandible *n* = 58	23 (0.40)	10 (0.17)	25 (0.43)	71 (0.61)	45 (0.39)
χ^2^ *p*-value φ Statistical test power	11.210 * 0.0243 0.235 0.77			11.351 * 0.0034 0.17 0.87	
	*IRF6 rs642961*				
	GG *n* (%)	AA *n* (%)	GA *n* (%)	G *n* (%)	A *n* (%)
Maxilla and mandible *n* = 106	74 (0.70)	3 (0.03)	29 (0.27)	177 (0.83)	35 (0.17)
Maxilla *n* = 38	26 (0.68)	0 (0.00)	12 (0.32)	64 (0.84)	12 (0.16)
Mandible *n* = 59	41 (0.69)	2 (0.03)	16 (0. 27)	98 (0.83)	20 (0.17)
χ^2^ *p*-value φ Statistical test power	1.412 0.8420 0.08 0.12			0.072 0.9648 0.01 0.05	
	*IRF6 rs861019*				
	AA *n* (%)	GG *n* (%)	AG *n* (%)	A *n* (%)	G *n* (%)
Maxilla and mandible *n* = 106	25 (0.24)	18 (0.17)	63 (0.59)	113 (0.53)	99 (0.47)
Maxilla *n* = 38	8 (0.21)	5 (0.13)	25 (0.66)	41 (0.54)	35 (0.46)
Mandible *n* = 59	18 (0.51)	10 (0.17)	31 (0.53)	67 (057)	51 (0.43)
χ^2^ *p*-value φ Statistical test power	2.025 0.7312 0.10 0.17			0.379 0.8276 0.03 0.08	
	*IRF6 rs4904210*				
	TT *n* (%)	CC *n* (%)	TC *n* (%)	T *n* (%)	C *n* (%)
Maxilla and mandible *n* = 106	43 (0.41)	13 (0.12)	50 (0.47)	136 (0.64)	76 (0.36)
Maxilla *n* = 38	12 (0.32)	5 (0.13)	21 (0.55)	45 (0.59)	31 (0.41)
Mandible *n* = 58	23 (0.40)	9 (0.16)	26 (0.45)	72 (0.62)	44 (0.38)
χ^2^ *p*-value φ Statistical test power	1.461 0.8336 0.085 0.13			0.605 0.7391 0.04 0.10	
	*IRF6 rs658860*				
	TT *n* (%)	CC *n* (%)	TC *n* (%)	T *n* (%)	C *n* (%)
Maxilla and mandible *n* = 106	74 (0.70)	3 (0.03)	29 (0.27)	177 (0.83)	35 (0.17)
Maxilla *n* = 37	26 (0.70)	0 (0.00)	11 (0.30)	63 (0.85)	11 (0.15)
Mandible *n* = 57	39 (0.68)	1 (0.02)	17 (0.30)	95 (0.83)	19 (0.17)
χ^2^ *p*-value φ Statistical test power	1.240 0.8715 0.08 0.12			0.129 0.9374 0.02 0.06	

*p*—statistical significance, χ^2^—Chi^2^ test result, *n*—number of subjects, *—significant statistical differences, φ—effect size.

**Table 5:** Comparison of allele and genotype frequencies for *MSX1* NM_002448.3:c.*6C>T (rs8670) and NM_002448.3:c.*276A>G (rs12532), and *AXIN2* NM_004655.3:c.148C>T (rs2240308) variants between study and control groups.

*MSX1 rs8670*	Control Group (*n* = 186)	Study Group (*n* = 202)	p_a_
CC (*n* = 228)	115 (61.8%)	113 (55.9%)	0.487 (χ² 1.44)
CT (*n* = 134)	60 (32.3%)	74 (36.6%)	
TT (*n* = 26)	11 (5.9%)	15 (7.4%)	
C 0.76	0.78	0.74	0.228 (χ² 1.46)
T 0.24	0.22	0.26	
HWE			
χ² = 1.07; pb = 0.301	χ² = 0.7; pc = 0.403	χ² = 0.35; pd = 0.554	
*MSX1 rs12532*	Control group (*n* = 186)	Study group (*n* = 202)	p_a_
AA (*n* = 209)	99 (53.2%)	110 (54.2%)	0.925 (χ² 0.16)
AG (*n* = 145)	71 (38.2%)	74 (36.5%)	
GG (*n* = 35)	16 (8.6%)	19 (9.4%)	
A 0.72	0.72	0.72	0.975 (χ² 0.001)
G 0.28	0.28	0.28	
HWE			
χ² = 1.8; pb = 0.180	χ² = 0.41; pc = 0.522	χ² = 1.56; pd = 0.212	
*AXIN2 rs2240308*	Control group (*n* = 186)	Study group (*n* = 202)	p_a_
AA (*n* = 113)	53 (28.5%)	60 (29.7%)	0.695 (χ² 0.73)
AG (*n* = 190)	95 (51.1%)	95 (47.0%)	
GG (*n* = 85)	38 (20.4%)	47 (23.3%)	
A 0.54	0.54	0.53	0.820 (χ² 0.05)
G 0.46	0.46	0.47	
HWE			
χ² = 0.09; pb = 0.764	χ² = 0.15; pc = 0.699	χ² = 0.62; pd = 0.431	

p_a_—values calculated for allele and genotype frequency comparisons between control and study groups; pb—values calculated for genotype Hardy–Weinberg proportions in all participants (control and study groups analyzed together); pc—values calculated for genotype Hardy–Weinberg proportions in control group; pd—values calculated for genotype Hardy–Weinberg proportions in study group; HWE—Hardy–Weinberg equilibrium.

**Table 6:** Number of impacted teeth in study group patients.

Number of Impacted Teeth	*n*	%
1	44	21.6
2	55	27.0
3	28	13.7
4 and more	77	37.7

## Data Availability

Data available on request due to restrictions—privacy or ethical.
